# Potentially suitable geographical area for *Pulsatilla chinensis* Regel under current and future climatic scenarios based on the MaxEnt model

**DOI:** 10.3389/fpls.2025.1538566

**Published:** 2025-05-14

**Authors:** Yanan Wu, Lanmeng Yan, Hongjian Shen, Rui Guan, Qianqian Ge, Ling Huang, Emelda Rosseleena Rohani, Jinmei Ou, Rongchun Han, Xiaohui Tong

**Affiliations:** ^1^ School of Pharmacy, Anhui University of Chinese Medicine, Hefei, China; ^2^ Institute of Systems Biology, Universiti Kebangsaan Malaysi, Bangi, Malaysia; ^3^ Joint Research Center for Chinese Herbal Medicine of Anhui of IHM, Anhui University of Chinese Medicine, Hefei, China; ^4^ School of Life Sciences, Anhui University of Chinese Medicine, Hefei, China; ^5^ Department of Research and Development, Functional Activity and Resource Utilization on Edible and Medicinal Fungi Joint Laboratory of Anhui Province, Jinzhai, China

**Keywords:** ArcGIS, ecological suitability zoning, future climate change, MaxEnt model, *Pulsatilla chinensis*

## Abstract

Climate change has significantly impacted the distribution patterns of medicinal plants, highlighting the need for accurate models to predict future habitat shifts. In this study, the Maximum Entropy model to analyze the habitat distribution of *Pulsatilla chinensis* (Bunge) Regel under current conditions and two future climate scenarios (SSP245 and SSP585). Based on 105 occurrence records and 12 environmental variables, precipitation of the wettest quarter, isothermality, average November temperature, and the standard deviation of temperature seasonality were identified as key factors influencing the habitat suitability for *P. chinensis*. The reliability of the model was supported by a mean area under the curve (AUC) value of 0.916 and a True Skill Statistic (TSS) value of 0.608. The results indicated that although the total suitable habitat for *P. chinensis* expanded under both scenarios, the highly suitable area contracted significantly under SSP585 compared to SSP245. This suggests the importance of incorporating climate change considerations into *P. chinensis* management strategies to address potential challenges arising from future ecosystem dynamics.

## 1 Introduction

As carbon emissions continue to rise, global warming has gradually become an irreversible trend. Under this influence, China’s climate and environment have undergone significant changes, including rising average annual temperatures and shifting precipitation patterns. Studies indicate that from 1951 to 2021, the average annual temperature in China increased by 0.26°C per decade, while annual precipitation rose by 5.5 mm per decade, accompanied by a northward shift in the primary rainy zone (Ren et al., 2021). The growth, development, and distribution status of plants are typically closely associated with climate (Huang et al., 2024; Ni and Vellend, 2024). Consequently, climate change will inevitably lead to some degree of alteration in species habitats (Li et al., 2019; Wouyou et al., 2022). For example, due to climate change, the range of *Gymnadenia conopsea* (L.) R. Br. is expected to contract and gradually shift to higher altitudes, such as those in Tibet and Yunnan (Cha et al., 2024). Similarly, the suitable area of *Paris polyphylla* var. *yunnanensis* (Franch.) Hand.-Mzt. is also expected to shrink significantly under future high greenhouse gas emission scenarios, with its center of mass shifting toward higher latitudes (Zhang et al., 2024). These cases exemplify the differentiated response mechanisms of medicinal plants to climate change, reflecting the two primary types of species habitat shifts: altitudinal and latitudinal migration. Therefore, understanding the interaction between climate change and plants is of great significance for plant resource conservation and exploitation.

In recent years, species distribution modeling (SDM) has become the method of choice for predicting changes in species’ habitats, which focuses on simulating areas of habitat distribution by analyzing known location information and ecological factors of species (Li et al., 2020; Wang et al., 2020; Low et al., 2021). Compared with models such as the Generalized Linear Model (GLM) (Hirzel et al., 2001) and DOMAIN model (Xia et al., 2024), the Maximum Entropy (Maxent) model is easy to operate, has higher prediction accuracy, and has been widely used in species distribution modeling studies (Glor and Warren, 2011; Fitzpatrick et al., 2013; Warren et al., 2014; Remya et al., 2015). By searching the Web of Science using “MaxEnt” and “species distribution” as keywords, we retrieved 906 and 1,145 publications in 2023 and 2024, respectively, which reflects the applicability of the MaxEnt model in plant ecology study. Although the maximum entropy model achieves high prediction accuracy with default parameters, its fixed feature combinations with regularization multipliers may lead to overfitting risk and do not apply to the prediction of all species distributions (Warren and Seifert, 2011; Moreno-Amat et al., 2015). In practice, parameters used for MaxEnt analysis will be adjusted in advance by using the R program to screen the optimal configuration based on environmental variables and distribution points. By this means, the model reliability can be improved to construct a more ecologically meaningful distribution model.

*Pulsatilla chinensis* (Bunge) Regel is a rhizomatous perennial herb of the buttercup family, whose rhizomes are widely used for clearing away heat and toxins, cooling the blood, and treating dysentery, with a particular specialty in removing toxins of dampness-heat and blood-heat from the gastrointestinal tract (Zhao et al., 2021). Pharmacological studies have reported that the active ingredients contained in *P. chinensis* extracts have demonstrated significant antitumor, anti-inflammatory, antibacterial, and antiviral effects (Cheng et al., 2008; Sun et al., 2010; Li et al., 2014). However, with the increasing demand for *P. chinensis* for medicinal purposes, its wild resources are gradually being depleted, leading to an increasing conflict between supply and demand. In the face of this situation, it is particularly urgent to strengthen the research on the distribution of *P. chinensis* resources and its ecological characteristics, which not only helps to scientifically and rationally formulate conservation measures but also provides strong support for the development of artificial cultivation technology and realizes the sustainable use of resources. However, there is a relative paucity of research on the resource distribution of *P. chinensis* and its habitat characteristics. Therefore, to investigate the habitat suitability dynamics and climate adaptation mechanisms of *P. chinensis*. In this study, we used the parameter-optimized MaxEnt model, combined with ArcGIS spatial analysis techniques, to systematically assess the distribution pattern of *P. chinensis* habitat suitability under the current climatic conditions, as well as under two future climate scenarios (SSP245 and SSP585) in the 2050s (2041–2060) and 2070s (2061–2080). Based on the variable contribution rankings and response curves of the model outputs, key climate factors driving the distribution of *P. chinensis* were identified, and their ecological threshold ranges were analyzed to reveal the adaptation boundaries of *P. chinensis* to climate change.

The aims of this study were as follows: (1) to predict the current distribution of *P. chinensis* into different suitability classes; (2) to analyze key variables affecting the growth of *P. chinensis*; and (3) to predict future changes in the habitat of *P. chinensis* in China under different carbon emission scenarios.

## 2 Materials and methods

### 2.1 Species distribution data of *P. chinensis*

**Figure 1:**
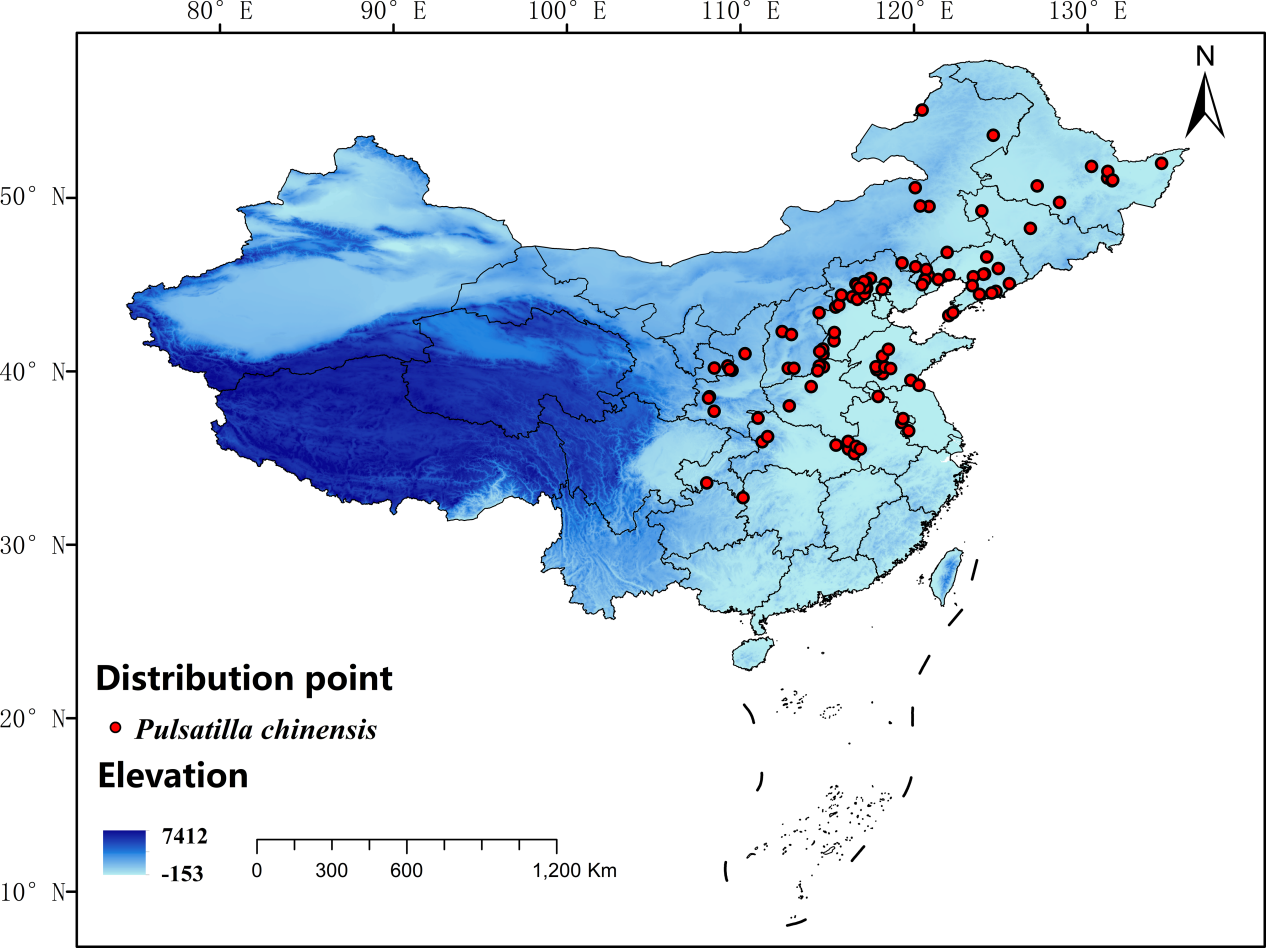
Records of *P. chinensis* in terms of distribution in China.

Information on the distribution points of *P. chinensis* in this study was obtained from the National Specimen Information Infrastructure (NSII, http://www.nsii.org.cn) and the Global Biodiversity Information Facility (GBIF, http://data.gbif.org). For distribution points lacking detailed latitude and longitude coordinates, geographic coordinates were obtained through Google Maps, and 133 coordinates were collected. Environmental variables exhibit significant spatial autocorrelation at short distances (Dormann et al., 2007). To prevent model overfitting caused by clustered distribution points, a 5 km × 5 km grid consistent with the resolution of the bioclimatic data (2.5 arc min) was generated by using a Spatially Rarefy tool in ArcGIS 10.8, with only one coordinate point retained in each grid. The 5-km spatial interval ensures both the independence of distribution points by representing different grid cells and avoids sampling too sparsely and missing key environmental assemblages. Eventually, 105 distribution points were retained for model construction (**Figure 1**).

### 2.2 Environmental data acquisition and processing

This study utilized climate data from the modern period (1970–2000) and two future periods (2041–2060 and 2061–2080). Ecosystem responses to climate change usually have a lag, and the selection of the 2050s and 2070s as time points for future scenarios can reflect changes in species distributions under the medium-term immediate response and the loss of habitat or the ability to adapt to new environments under the forward cumulative effect. The climate data included in this study include monthly precipitation, January-December mean temperature, and 19 bioclimatic variables, which were obtained from WorldClim (http://www.worldclim.org) with a spatial resolution of 2.5 arcminutes (about 5 km). This resolution achieved a good balance between data accuracy and computational efficiency. In addition, ArcGIS was used to extract slope and aspect direction from the digital elevation model provided by WorldClim, which characterize the degree of inclination and orientation of the ground surface, respectively. Data for soil were obtained from the Harmonized World Soil Database (HWSD, http://vdb3.soil.csdb.cn/). Future bioclimatic variables were accessed from the WorldClim database, which integrates datasets derived from the BCC-CSM2-MR climate model under the Sixth Coupled Model Intercomparison Project (CMIP6) (Wu et al., 2019). BCC-CSM2-MR is suitable for modeling the Chinese climate and provides relatively accurate forecast data (Fick and Hijmans, 2017). This study modeled the potential future fitness zones of *P. chinensis* under two typical concentration emission scenarios, SSP245 and SSP585. SSP 245 represents a development pattern with a moderate level of greenhouse gas emissions, while SSP 585 is a development pattern with fossil fuels as the main source of energy, reflecting higher greenhouse gas emissions (Zhang et al., 2019).

To avoid the potential impact of multicollinearity on model stability, this study first excluded variables with zero contribution rate using the MaxEnt 3.4.4 to ensure that candidate variables were potentially ecologically driven (Shi et al., 2021; Guo et al., 2023). The Pearson correlation coefficients of the variables screened by MaxEnt were then calculated using Statistical Product and Service Solutions (SPSS, version 27.0), and for the group of highly correlated variables (|*r*| ≥ 0.8), those with high contributions were retained (Zhang et al., 2022). Finally, 12 variables were obtained for model analysis (**Table 1**).

**Table 1:** Variables used for modeling.

Variable	Environmental factor
Aspect	Aspect (°C)
Bio2	Mean diurnal range (°C)
Bio3	Isothermality (%)
Bio4	Standard deviation (SD) of temperature seasonality (°C)
Bio15	Precipitation seasonality (mm)
Bio16	Precipitation of the wettest quarter (mm)
Elev	Elevation (°)
Pre1	January precipitation (mm)
Pre12	December precipitation (mm)
Slope	Slope (°)
T_clay	Percentage clay respectively in the topsoil (%)
Tmean11	Average November temperature (°C)

### 2.3 Species distribution modeling

The distribution data of *P. chinensis*, along with associated environmental variables, were processed using the MaxEnt model. Logistic output was selected, and the random test percentage was configured to 25%, reserving 75% of the dataset for training purposes (Amiri et al., 2022). The model was set to run for a maximum of 500 iterations with 10,000 pseudo-absence points, and cross-validation was conducted ten times. The use of 10,000 background points is usually sufficient to characterize the environmental space of the study area. When there are fewer species distribution points, appropriately increasing the number of background points can help reduce potential sampling bias (Barber et al., 2022). To determine the importance of each variable, Jackknife tests were employed, followed by an evaluation using the ROC curve. The ROC curve, a widely recognized tool for assessing binary classifiers, illustrates the balance between true positive and false positive rates. Model performance was quantified through the area under the curve (AUC), with the following interpretation: 0.50–0.60 indicates failure, 0.60–0.70 poor, 0.70–0.80 fair, 0.80–0.90 good, and 0.90–1.00 very good (Phillips et al., 2006).

To comprehensively assess the predictive performance of the model, we also chose the True Skill Statistic (TSS) to verify the reliability of the results. TSS is an indicator that assesses the predictive performance of species distribution models, which effectively corrects for sample imbalance bias in species distribution data by integrating sensitivity and specificity (Allouche et al., 2006). The value of TSS ranges from − 1 to 1, and the closer to 1 the better the prediction results; when the value of TSS is located between 0.6 and 1, the prediction ability can be considered good. The calculation of TSS values in this study was done in R 4.4.1.

### 2.4 Optimization of the model

The MaxEnt model regularization level is governed by two critical parameters: the regularization multiplier (RM) and feature combinations (FC) (Radosavljevic and Anderson, 2014). The feature classes available include linear (L), quadratic (Q), hinge (H), product (P), and threshold (T) (Elith et al., 2011). To identify the optimal parameter settings, RM values were incrementally varied from 0.5 to 4.0 in steps of 0.5, and tested with six combinations of feature classes: L, LQ, H, LQH, LQHP, and LQHPT. The Akaike Information Criterion Correction (AICc) is an important metric for assessing model fit (Muscarella et al., 2014). In this study, we used the ENMeval package for R to calculate the AICc values of the MaxEnt models for different parameter settings. Delta AICc is a metric used in model selection to measure the relative difference between candidate models and the optimal model. The delta AICc value is obtained by calculating the AICc values and then subtracting the AICc of each model from the smallest AICc value among all candidate models. Larger values of delta AICc are less likely to serve as the best approximation of the model in the candidate set. Models with delta AICc ≤ 2 have substantial support, while models with delta AICc > 10 have essentially no support (Burnham and Anderson, 2004). For our study on *P. chinensis*, the model with delta AICc = 0 was finally chosen.

### 2.5 Data processing

The grading and visualization of *P. chinensis* suitable areas were achieved by loading the mean results of the model output into ArcGIS software. Suitability probability classes were classified using the natural breakpoint method, which determines the optimal classification threshold based on the distributional characteristics of the data itself. There were four categories: highly suitable (0.5–1.0), moderately suitable (0.3–0.5), lowly suitable (0.1–0.3), and unsuitable (0.0–0.1). Dynamic changes in center-of-mass trajectories better reflect the spatial and temporal reconfiguration characteristics of the distribution pattern than area fluctuations. Therefore, in this study, while quantifying the areas with different suitability classes, we used the spatial statistical tools in ArcGIS to transform the distribution of *P. chinensis* suitable areas in each period into representative centroids (Brown, 2014; Guo et al., 2023; Wang et al., 2024), which reflected the overall spatial positioning of *P. chinensis* suitability zones.

## 3 Results and analysis

### 3.1 Model optimization accuracy evaluation

**Figure 2:**
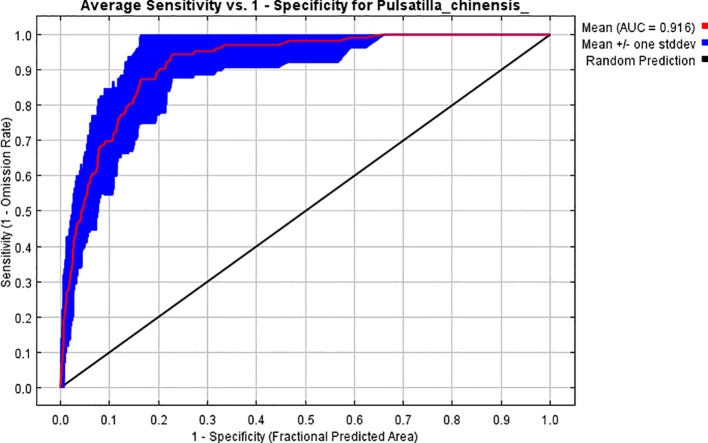
ROC curves of the MaxEnt model for *P. chinensis*.

The distribution area of *P. chinensis* was predicted based on 105 occurrence records and 12 environmental variables. When running the model with the MaxEnt default parameters (RM = 1, FC = LQH), delta AICc = 317.77, it indicated a risk of overfitting. Nevertheless, adopting the adjusted parameters (RM = 2.5, FC = LQHPT), delta AICc = 0, demonstrated that the predictive accuracy of the model was better than the default model for this combination of parameters. The results showed that the mean AUC of the model under the optimized parameters was 0.916 and the mean TSS was 0.608, both of which further confirmed the reliability of the model results (**Figure 2**).

### 3.2 Distribution of *P. chinensis* concerning environmental variables

**Figure 3:**
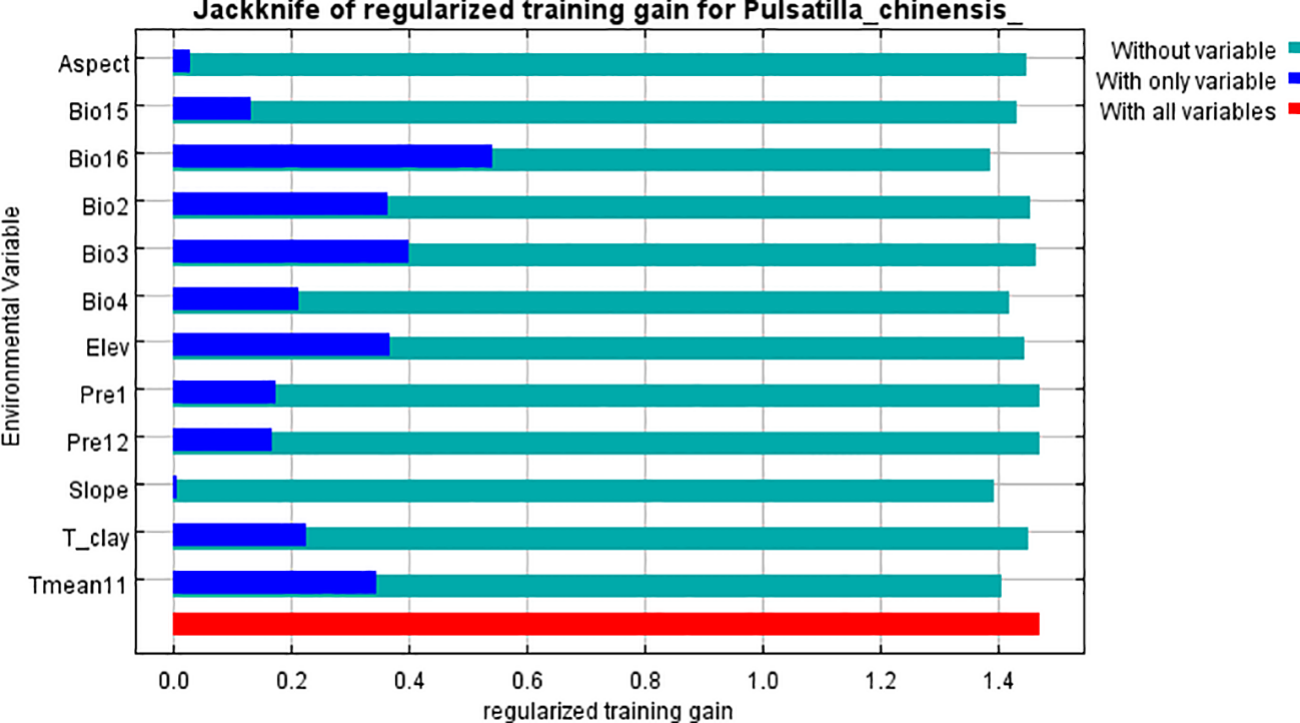
Jackknife test of environmental variables for *P. chinensis*.

Model results indicate that Precipitation of the wettest quarter, Isothermality, Average November temperature, and Standard deviation of temperature seasonality collectively contributed 83.3% to the habitat suitability of *P. chinensis*. The precipitation of the wettest quarter ranked as the most influential variable, contributing 32.7% (**Table 2**). The Jackknife test revealed that Precipitation of the wettest quarter, Isothermality, and Mean diurnal range had significant impacts on the survival of *P. chinensis*. This highlights precipitation and temperature as key determinants of *P. chinensis* habitat suitability (**Figure 3**).

**Table 2:** Contribution and significance of variables.

Variable	Percent contribution (%)	Permutation importance (%)
Bio16	32.7	25.4
Tmean11	19	12.4
Bio3	17.9	0.9
Bio4	13.7	15.5
Slope	6.6	8.3
Bio15	3.9	6.4
Elev	2.1	17
Aspect	1.4	1.8
Bio2	1.4	10
T_clay	1.1	1.9
Pre1	0.1	0.3
Pre12	0.1	0.2

**Figure 4:**
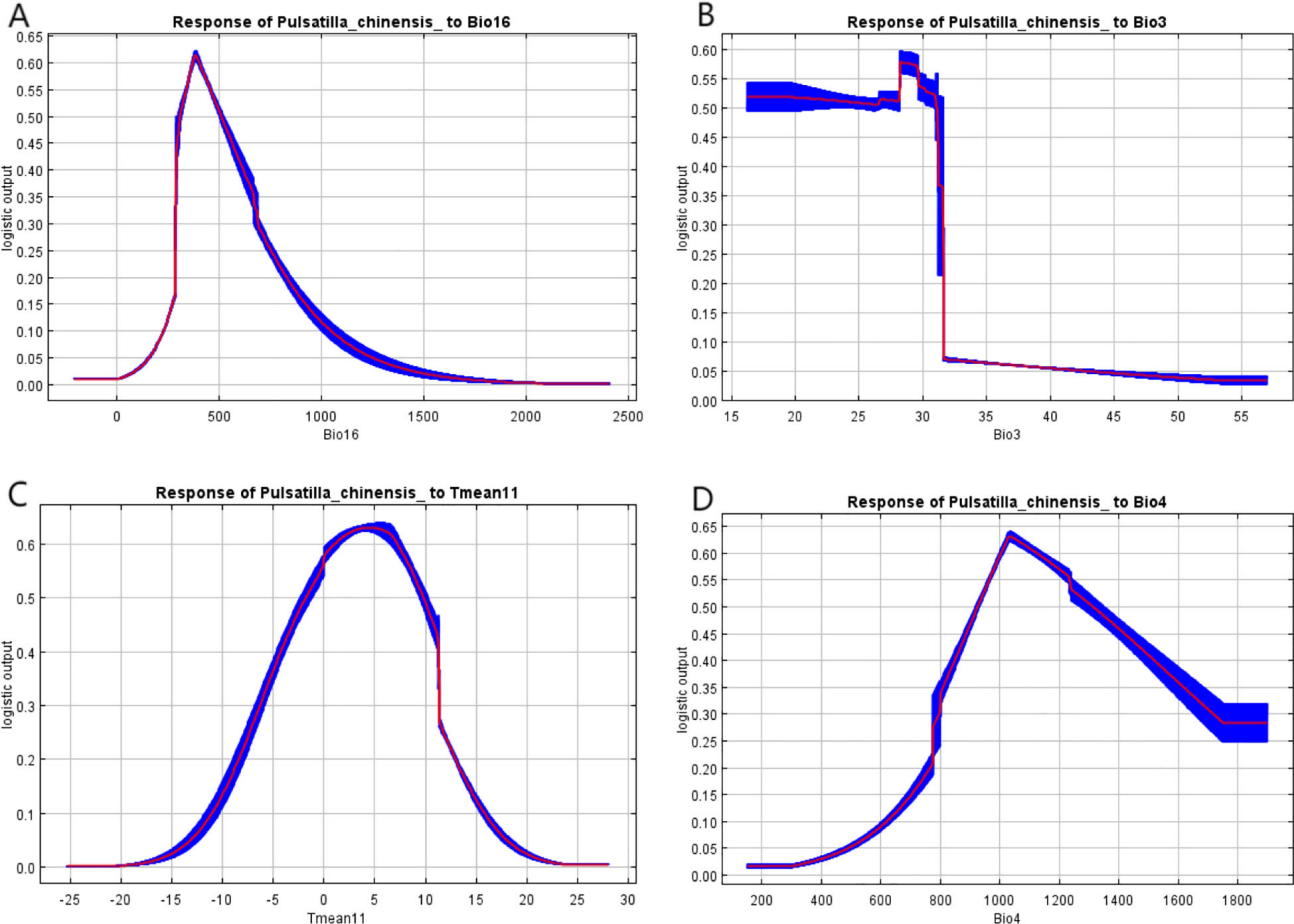
Response curves for the critical variables: precipitation of the wettest quarter **(A)**, isothermality **(B)**, average November temperature **(C)**, and standard deviation of temperature seasonality **(D)**.

Response curves were generated by univariate marginal effects analysis in the MaxEnt model, reflecting each variable’s marginal contribution to species habitat suitability while holding other variables constant. In this study, response curves for the four variables with the highest contributions were specifically analyzed using a threshold probability of occurrence greater than 0.50 (Liu et al., 2005; Xu et al., 2023b). The presence of *P. chinensis* was most likely when the precipitation of the wettest quarter ranged from 307.5 to 506.9 mm. *P. chinensis* grows best in this region when isothermality values range from 16.2% to 31.1%. The likelihood of *P. chinensis* presence exceeded 50% when the average November temperature ranged from − 1.7°C to 9.7°C. Additionally, *P. chinensis* exhibited higher survival rates when the standard deviation of temperature seasonality ranged from 9.3°C to 13.1°C (**Figure 4**).

### 3.3 Current distribution of *P. chinensis* in China

**Figure 5:**
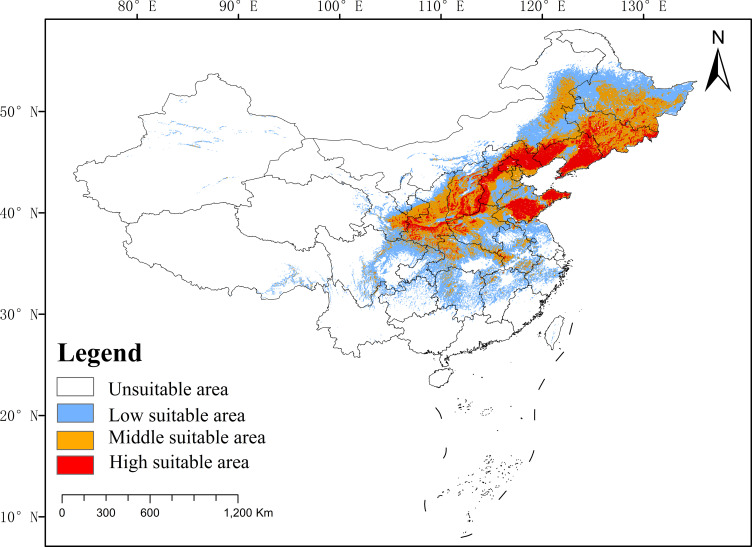
Distribution of *P. chinensis* in China.

The distribution of *P. chinensis* is primarily concentrated in northern and eastern China, with a total suitable area estimated at 262.78 × 10^4^ km². The highly suitable zone covers 47.60 × 10^4^ km², accounting for 18.12% of the total suitable area, and is primarily distributed across Liaoning, Beijing, Shandong, Hebei, and Shanxi. The moderately suitable zone spans 84.02 × 10^4^ km², representing 31.98% of the total suitable area. It surrounds the highly suitable zone and extends into regions such as Inner Mongolia, Jilin, and Shaanxi. The low suitability zone occupies 131.14 × 10^4^ km², making up 49.91% of the total suitable area. It is primarily located around the moderately suitable zone, spanning central, eastern, and southwestern China (**Figure 5**).

### 3.4 Distributional changes of *P. chinensis* under future climates

Projections suggest an overall expansion of suitable habitat for *P. chinensis* under both future emission scenarios. The SSP245 scenario predicts a more pronounced increase in suitable habitat compared to SSP585 (**Table 3**).

**Table 3:** Distribution of *P. chinensis* under current and future scenarios (× 10^4^ km^2^).

Scenarios	Unsuitable	Low suitable	Middle suitable	High suitable	Total suitable
Current	666.4479	131.1424	84.0277	47.6093	262.7794
SSP245 2050S	632.9549	128.2292	88.7691	112.5365	329.5348
SSP245 2070S	638.5816	139.0122	65.8020	119.0938	323.9080
SSP585 2050S	676.3507	115.7135	102.3681	68.0572	286.1388
SSP585 2070S	680.2292	109.0365	105.7917	67.4322	282.2604

**Figure 6:**
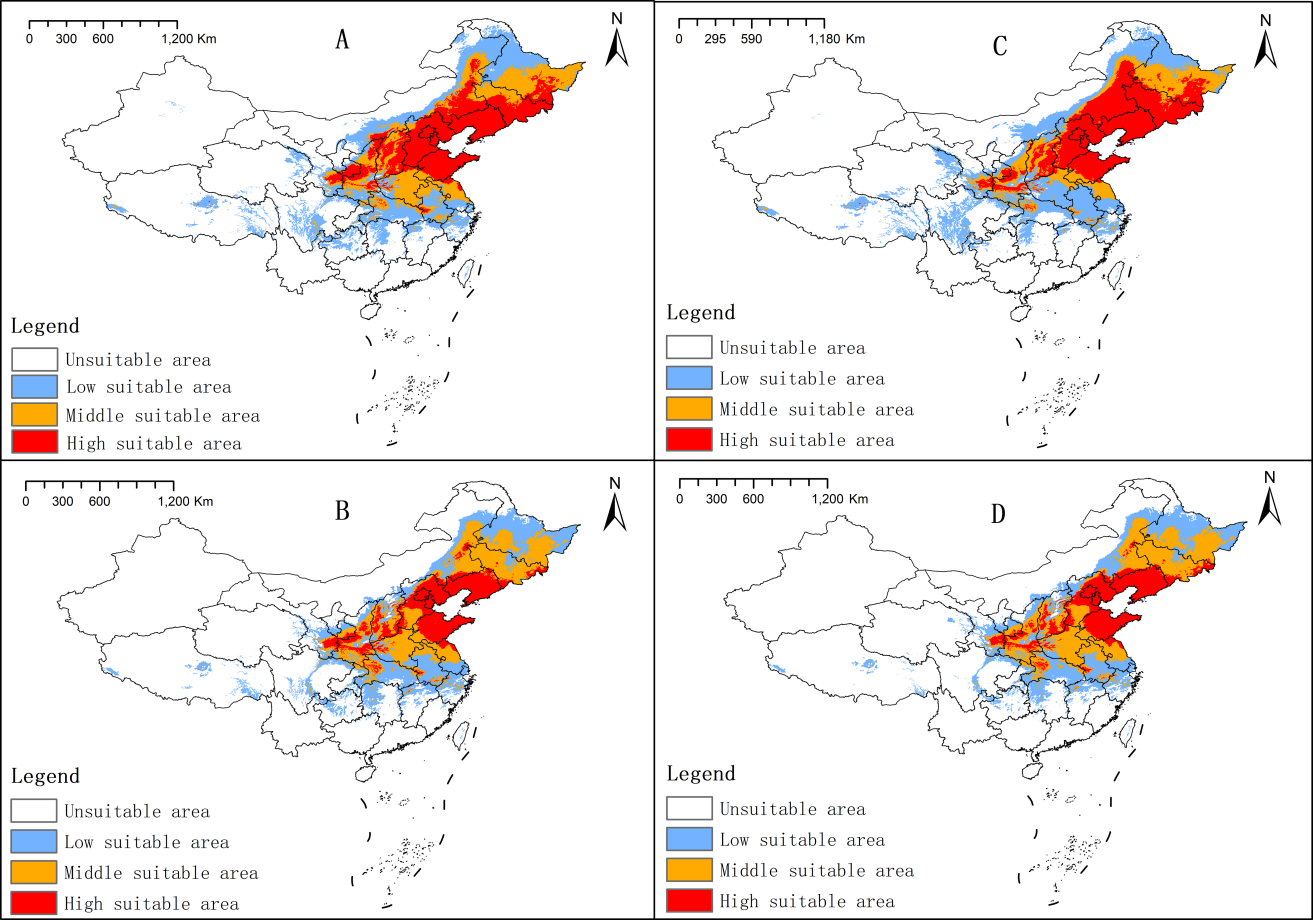
Changes in *P. chinensis* suitability areas under future climate conditions. **(A)** 2041–2060, SSP 245; **(B)** 2041–2060, SSP 585; **(C)** 2061–2080, SSP 245; and **(D)** 2061–2080, SSP 585.

Between 2041 and 2060, the total suitable area under SSP245 increased by 25.40% relative to the current period, with significant expansion in highly suitable areas (**Figure 6A**). Key suitable regions included Shandong, Hebei, Beijing, Tianjin, Liaoning, Jilin, Heilongjiang, Inner Mongolia, Shanxi, Shaanxi, and Gansu. Under SSP585, the total suitable area increased by 8.89% compared to the current period (**Figure 6B**). While moderately suitable areas expanded, highly suitable areas experienced a relative decline.

Between 2061 and 2080, the SSP245 scenario projected a 23.26% increase in total suitable area, with further expansion in highly suitable regions, particularly in Jilin, Heilongjiang, and Inner Mongolia (**Figure 6C**). The overall distribution pattern remained consistent with projections for 2041–2060 under the SSP245 scenario. Under SSP585, the total suitable area increased by 7.41% compared to the current period, with a slight decrease relative to the 2041–2060 projections. The distribution pattern remained largely unchanged (**Figure 6D**). According to ArcGIS, the threshold for a highly suitable area is greater than 0.5. To present the data from another perspective, we redrew the highly suitable areas with an adjusted threshold greater than 0.8, considering current (**Supplementary 1**) and future scenarios (**Supplementary 2**).

### 3.5 Changes in the center of mass of *P. chinensis* at different periods

**Figure 7:**
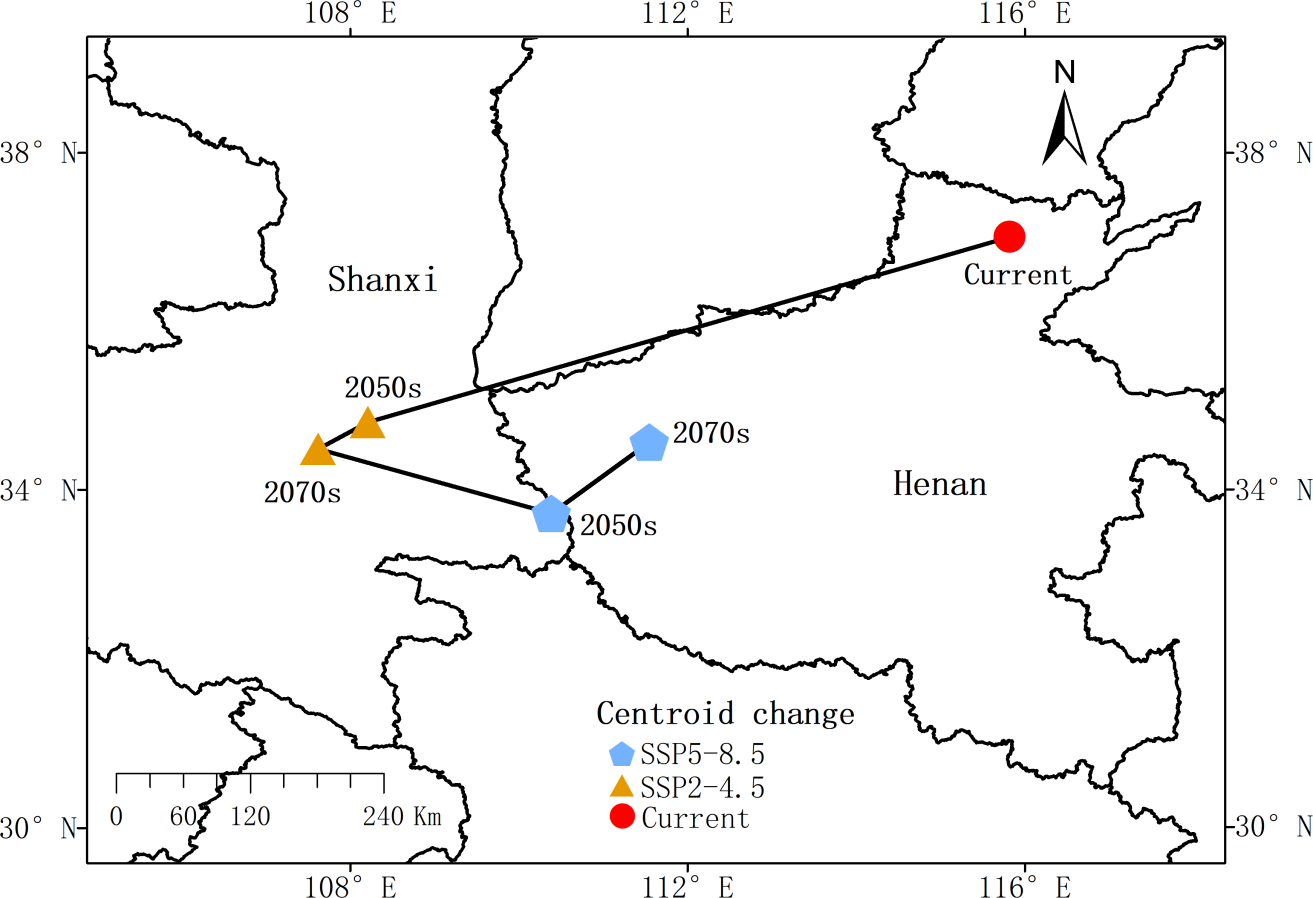
Map of the center of gravity shifts of *P. chinensis* suitable areas under future climate scenarios.

The center of mass of *P. chinensis* exhibits slight variations under different emission scenarios (**Figure 7**). Under the SSP245 scenario, the center of mass of *P. chinensis* is projected to shift westward from Tangyin County, Anyang City, Henan Province, to Lintong District, Xi’an City, Shaanxi Province, covering a distance of approximately 501.28 km between 2041 and 2060. It is expected to move further to Chang’an District, Xi’an City, Shaanxi Province, with an additional shift of about 544.32 km between 2061 and 2080.

Under the more severe SSP585 scenario, the center of mass is projected to shift southwestward to Shangnan County, Shangluo City, Shaanxi Province, over a distance of approximately 420.01 km between 2041 and 2060. Subsequently, it is expected to move southeastward to Luoning County, Luoyang City, Henan Province, with an additional shift of about 322.98 km between 2061 and 2080. Overall, the center of mass of *P. chinensis* tends to shift southwestward. Notably, the SSP245 scenario predicts a longer shift distance compared to SSP585, primarily spanning Shaanxi and Henan provinces.

## 4 Discussion

### 4.1 Optimized performance of MaxEnt

Previous studies have demonstrated that optimizing model parameters can reduce complexity and minimize error rates during operations (Shi et al., 2024). In this study, the ENMeval toolkit in R was employed to optimize the initial MaxEnt parameters, utilizing filtered *P. chinensis* distribution data and key ecological factors, including climate, topography, and soil. The adjusted parameters are RM = 2.5 and FC = LQHPT. The results demonstrate that the mean value of AUC was 0.916 and the mean value of TSS was 0.608, both of which exceeded the ecological model validity threshold, which indicates the reliability of the model results.

### 4.2 Major environmental variables affecting the distribution of *P. chinensis*

The results showed that precipitation of the wettest quarter (Bio16), isothermality (Bio3), average November temperature (Tmean11), and standard deviation of temperature seasonality (Bio4) were the key environmental variables affecting the geographical distribution of *P. chinensis* with a total contribution of 83.3%. This shows that temperature and precipitation play a central role in regulating the growth rhythm and distribution pattern of *P. chinensis*. By analyzing the response curves, it was found that Bio16 at 307.5–506.9 mm, Bio3 at 16.2%–31.1%, Tmean11 at − 1.7°C or to 9.7°C, and Bio4 at 9.3°C—13.1°C were suitable for *P. chinensis* growth.

Temperature and precipitation are the main biological determinants of plant growth status, and there are differences in water and temperature requirements between plants, with drought and high temperatures reducing the efficiency of water uptake by plants (Punyasena et al., 2008; Bradie and Leung, 2017). The *P. chinensis* tends to grow in light, moderately rainy environments, is tolerant of cold, and wilts easily under hot, rainy conditions (Xu et al., 2023a). The *P. chinensis* has a shallow root system and has a significantly higher water requirement during its growth spurt. Adequate precipitation not only increases soil water content to promote root development but also provides the necessary conditions for photosynthesis and reproductive development, while excessive precipitation may lead to waterlogging of the soil, which induces diseases such as root rot and thus inhibits the expansion of its population (Fang et al., 2021). Bio3 and Bio4 together reflect the magnitude of intra-annual temperature fluctuations. As a temperate plant, *P. chinensis* is sensitive to temperature changes that affect dormancy lifting and growth initiation. A small diurnal temperature difference helps to avoid extreme temperature stress and maintain normal photosynthesis and metabolic activities (Khodorova and Boitel-Conti, 2013). Moderate seasonal variation in temperature is conducive to the successful completion of the entire process of growth, differentiation, reproduction, and entry into dormancy of *P. chinensis* between seasons. The *P. chinensis* is more likely to establish stable populations in more humid climates with less temperature fluctuation. Before entering winter dormancy, *P. chinensis* still requires some degree of nutrient accumulation and root activity. Suitable November temperatures help it to complete the synthesis and storage of nutrients, laying the foundation for winter dormancy and emergence in the following spring. If the temperature is too low, it may lead to passive early dormancy, which can negatively affect nutrient accumulation and growth in the following year. It is worth noting that there may be synergistic effects between these key variables. For example, in areas with moderate temperature seasonality and favorable November temperatures, *P. chinensis* can fully accumulate nutrients before winter. However, if temperature seasonality is too high, even if the late fall temperatures are suitable, the species’ growth may still be hindered by sharp temperature fluctuations throughout the year.

The state of plant growth and distribution is the result of the synergistic effect of multidimensional ecological factors (Zhou et al., 2025). Although climatic variables are often shown to be the dominant drivers, factors such as soil physicochemical properties and geomorphological and hydrological features likewise shape species’ ecological niche boundaries (Benning and Moeller, 2021). Therefore, when formulating resource maintenance strategies, priority can be given to monitoring the dynamic thresholds of key climatic parameters while establishing a multifactor synergistic early warning mechanism to safeguard the ecological adaptability of populations and ultimately realize the sustainable use of resources.

### 4.3 Changes in the suitability distribution of *P. chinensis*

Previous studies have identified northeastern China—comprising Jilin, Liaoning, Hebei, Shandong, and Henan provinces—as the primary distribution area of *P. chinensis*. This is consistent with the predictions made in this study. Analysis of habitat changes indicates that the total suitable area for *P. chinensis* shows a general increasing trend under both SSP245 and SSP585 scenarios. Under SSP245, *P. chinensis* is projected to experience the most significant increase in suitable area, with expansions of 25.40% (2050s) and 23.26% (2070s) compared to the current period. Under SSP585, the suitable area continues to expand relative to the current period but is reduced by 13.17% (2050s) and 12.86% (2070s) compared to SSP245.

This suggests that although warming may have expanded the habitat of *P. chinensis* to some extent, the stability of its habitat is more challenged under more extreme warming scenarios. The relatively mild temperature increases in the SSP245 scenario, coupled with a moderate increase in precipitation, may provide a more balanced mix of water and heat for *P. chinensis*, which is conducive to the maintenance of its growth rhythm and life-cycle processes. The SSP585 scenario has a greater temperature increase, which may lead to high-temperature stress and water deficit, limiting population expansion. In addition, as a perennial herb, seed germination and seedling emergence of *P. chinensis* are extremely sensitive to spring temperatures and soil moisture, and climate extremes will affect population renewal and dispersal. Climate change not only affects the extent of species’ habitats but also drives the displacement of their centers of distribution (Cheng et al., 2008). When climatic conditions are outside the appropriate range for a species to survive, changes in its geographical distribution will follow (Campos et al., 2023). By calculating the distance of the center of mass movement in different periods, it was found that the center of mass of *P. chinensis* showed a tendency to shift to the southwestern direction. The study suggests that a warming climate will drive some species to higher altitudes and latitudes, causing them to expand and contract (Zu et al., 2021; Nuñez et al., 2023). However, since species have different physiological characteristics, their responses in the face of climate change are not entirely uniform. This study demonstrates that the habitat of *P. chinensis* shifted towards lower rather than higher latitudes. Species shifts toward lower latitudes often result from deteriorating habitat conditions driven by climate change, compounded by geographical anomalies (Sun et al., 2020). Variations in migration direction and distance are often influenced by the magnitude of climate warming (Poggio et al., 2018). Although temperatures are rising in the northern high latitudes, they may be unfavorable to the expansion of *P. chinensis* due to ecological constraints such as soil, light, or phenology. The southwestern region is characterized by mountains and plateaus, which may provide diverse microclimatic conditions for *P. chinensis*. Therefore, *P. chinensis* may maintain its adaptation to its ecological niche by migrating to lower latitudes but higher altitudes.

Changes in the distribution areas of species are the result of a combination of factors, including climate change, ecological changes, human activities, species competition, and geographical factors. This phenomenon reflects the dynamic adaptive capacity of plants to environmental changes and also reminds us to pay attention to the potential impacts of ecosystem changes on biodiversity. In response to the dynamic change of species’ habitats under the high carbon emission scenario, we can prioritize the establishment of climate-adapted protected areas in the expansion areas of habitats, carry out habitat restoration in the contraction areas, and layout relocation and protection corridors along the migration path of the center of mass of the habitats to connect the fragmented habitats and reduce the risk of migration and extinction.

### 4.4 Limitations of the study

The present study has several limitations that should be considered when predicting the distribution pattern of *P. chinensis*. First, since the model was constructed using only distribution records from within China, it may not fully capture the physiological tolerance range of the species (Feeley and Silman, 2011). Second, although parameter optimization was performed using the ENMeval toolkit, model performance may still be influenced by the choice of feature combinations. Different climate models exhibit systematic biases due to variations in physical processes and parameterization schemes (Tett et al., 2022), which can reduce the accuracy of their reproduction of historical climate states and the reliability of their future projections (Zhu and Yang, 2020). The BCC-CSM2-MR model used in this study shows significant improvements in simulating seasonal mean precipitation in East Asia; however, its temperature simulation is less accurate, with larger errors in inter-monthly variations compared to the BCC-CSM1.1m model (Li et al., 2023). In addition, the MaxEnt model has some inherent limitations (Feng et al., 2019; Schnase et al., 2021). It assumes a static environmental response and does not account for dynamic adaptive processes, such as phenotypic plasticity. The model also presumes that species can freely migrate to all suitable habitats, but in reality, seed dispersal distance limitations and human land-use barriers can significantly constrain the actual dispersal ability of species (Mou et al., 2025). Furthermore, biotic interactions and interspecies competition also play essential roles in species distribution (Hooper et al., 2005; Wisz et al., 2013). These factors may introduce a directional bias in the predictions, potentially leading to an overestimation of the expansion trend of suitable areas.

Future research suggestions include the following: (1) Integrating transboundary distribution data with functional trait parameters to construct ecological niche models that cover the full range of species; (2) using CMIP6 multimodel ensembles and downscaling techniques to quantify climate sensitivity under different emission scenarios, thereby reducing the uncertainty of regional projections; and (3) coupling individual-based and adaptive dynamic models to simulate the role of phenotypic plasticity and gene flow on the boundary expansion. These measures will aid in identifying credible expansion areas and provide scientific support for risk assessment in conservation planning.

## 5 Conclusion

The primary distribution of *P. chinensis* is currently concentrated in northern and eastern China, including the provinces of Liaoning, Hebei, and Shandong. Future climate projections suggest a general expansion of suitable habitats for *P. chinensis*, with the most significant increase occurring under the SSP245 scenario. Among the 12 variables analyzed, precipitation of the wettest quarter, isothermality, average November temperature, and the standard deviation of temperature seasonality emerged as the most influential factors affecting the growth of *P. chinensis*. The center of mass of *P. chinensis* habitats exhibited a southwestward shift, primarily driven by climate change. This study analyzes the spatial response patterns of *P. chinensis* habitat distribution under climate change and provides scientific support for its habitat adaptation, cultivation, and sustainable resource utilization.

## Data Availability

The original contributions presented in the study are included in the article/**Supplementary Material**. Further inquiries can be directed to the corresponding authors.
