# Supplementary material for: Potentially suitable geographical area for Pulsatilla chinensis Regel under current and future climatic scenarios based on the MaxEnt model
Source: Front Plant Sci. 2025 May 14;16:1538566. doi: 10.3389/fpls.2025.1538566 (PMC12116669; doi:10.3389/fpls.2025.1538566)
Supplement: Supplementary file 1 [file DataSheet1.docx]

**Supplementary material 1.** Distribution areas with current fitness thresholds greater than 0.8.





**Supplementary material 2.** Distribution areas with future fitness thresholds greater than 0.8. (A) 2041-2060, SSP 245; (B) 2061-2080, SSP 245; (C) 2041-2060, SSP 585; (D) 2061-2080, SSP 585**.**
